# The role of tyrosine hydroxylase as a key player in neuromelanin synthesis and the association of neuromelanin with Parkinson’s disease

**DOI:** 10.1007/s00702-023-02617-6

**Published:** 2023-03-20

**Authors:** Toshiharu Nagatsu, Akira Nakashima, Hirohisa Watanabe, Shosuke Ito, Kazumasa Wakamatsu, Fabio A. Zucca, Luigi Zecca, Moussa Youdim, Maximilian Wulf, Peter Riederer, Johannes M. Dijkstra

**Affiliations:** 1 Center for Research Promotion and Support, Fujita Health University, Toyoake, Aichi Japan; 2 Department of Physiological Chemistry, School of Medicine, Fujita Health University, Toyoake, Aichi Japan; 3 Department of Neurology, School of Medicine, Fujita Health University, Toyoake, Aichi Japan; 4 Institute for Melanin Chemistry, Fujita Health University, Toyoake, Aichi Japan; 5 Institute of Biomedical Technologies, National Research Council of Italy, Segrate (Milan), Italy; 6 Technion-Rappaport Family Faculty of Medicine, Haifa, Israel; 7 Department of Biology, Yonsey World Central University, Seoul, South Korea; 8 Medical Proteome-Analysis, Center for Protein Diagnostics (PRODI), Ruhr-University Bochum, Bochum, Germany; 9 Medizinisches Proteom‑Center, Medical Faculty, Ruhr-University Bochum, Bochum, Germany; 10 Clinic and Polyclinic of Psychiatry, Psychosomatics and Psychotherapy, University Hospital, Würzburg, Germany; 11 Department and Research Unit of Psychiatry, Syddansk University, Odense, Denmark; 12 Center for Medical Science, Fujita Health University, Toyoake, Aichi Japan

**Keywords:** Dopamine, Locus coeruleus, Melanin, Neuromelanin, Norepinephrine, Parkinson’s disease, Substantia nigra, Tyrosinase, Tyrosine hydroxylase

## Abstract

The dark pigment neuromelanin (NM) is abundant in cell bodies of dopamine (DA) neurons in the substantia nigra (SN) and norepinephrine (NE) neurons in the locus coeruleus (LC) in the human brain. During the progression of Parkinson’s disease (PD), together with the degeneration of the respective catecholamine (CA) neurons, the NM levels in the SN and LC markedly decrease. However, questions remain among others on how NM is associated with PD and how it is synthesized. The biosynthesis pathway of NM in the human brain has been controversial because the presence of tyrosinase in CA neurons in the SN and LC has been elusive. We propose the following NM synthesis pathway in these CA neurons: (1) Tyrosine is converted by tyrosine hydroxylase (TH) to L-3,4-dihydroxyphenylalanine (L-DOPA), which is converted by aromatic L-amino acid decarboxylase to DA, which in LC neurons is converted by dopamine β-hydroxylase to NE; (2) DA or NE is autoxidized to dopamine quinone (DAQ) or norepinephrine quinone (NEQ); and (3) DAQ or NEQ is converted to eumelanic NM (euNM) and pheomelanic NM (pheoNM) in the absence and presence of cysteine, respectively. This process involves proteins as cysteine source and iron. We also discuss whether the NM amounts per neuromelanin-positive (NM^+^) CA neuron are higher in PD brain, whether NM quantitatively correlates with neurodegeneration, and whether an active lifestyle may reduce NM formation.

## Introduction

Parkinson’s disease (PD) is a human-specific, aging-related, progressive neurodegenerative disorder. PD is the second most common neurodegenerative disease after Alzheimer’s disease. The main symptoms of PD are movement disorders, such as tremor, bradykinesia, rigidity, and postural instability, as well as non-motor ones, including anosmia, constipation, insomnia, rapid eye movement (REM)-sleep behavior disorder (RBD), anxiety, depression, fatigue, and cognitive impairment. Only a few percent of cases are familial PD and have a strong genetic component. Most PD is sporadic without a familial history. The main degenerating neurons in PD are dopamine (DA) neurons in the substantia nigra (SN) and norepinephrine (NE) neurons in the locus coeruleus (LC) (reviewed by Nagatsu et al. 2022). Because dopaminergic SN neurons innervate the striatum, major PD symptoms derive from the reduction of DA levels in the striatum which causes difficulties to engage in motor activities.

There are two main histological hallmarks of PD in the SN and LC (Greenfield and Bosanquet 1953; Bernheimer et al. 1973; Braak et al. 2003; Nagatsu et al. 2022): (1) reduction in neuromelanin (NM), which is a black-brown pigment in DA neurons and NE neurons; and (2) an accumulation of Lewy bodies, which are aggregates that contain α-synuclein as the main protein component, in both these types of catecholamine (CA) neurons. Fibrillar and prefibrillar oligomers of α-synuclein produced by misfolding are presumed to be neurotoxic and to cause CA neuron death (Mehra et al. 2019; Riederer et al. 2019). In contrast, the pathophysiology associated with the decreases in NM amounts in SN and LC remains not well understood (Hirsch et al. 1988, 1989; Fasano et al. 2006; Zucca et al. 2017; Nagatsu et al. 2022). In Parkinsonian SN and LC, in parallel with the marked reductions in NM in these tissues, NM-containing CA neurons were found to preferentially degenerate (Mann and Yates 1983; Hirsch et al. 1988, 1989). This suggests that NM is involved in neurodegeneration and CA neuron death. That would agree with the finding that human NM, upon injection into rat brain SN in vivo, produces inflammation and degeneration of DA neurons (Zecca et al. 2008a; Zhang et al. 2011). However, there remains discussion on how, per cell, the concentrations of NM in dying versus surviving CA neurons compare to each other in PD subjects and to non-PD controls. A major reason for NM not being well understood is that elucidation of the chemical structures of NM has been difficult owing to the small amounts available for research only from postmortem human brains and low solubility of NM. Along with these difficulties, the biosynthetic pathways are complicated and some steps still need to be investigated. This review provides a historic overview of important observations on the biosynthetic pathways of NM and addresses some issues of how NM amounts are associated with PD. The review is based on an oral presentation by Toshiharu Nagatsu and the ensuing discussion with an audience of experts, with the idea that this would highlight important questions in the field (see the acknowledgments). That discussion is summarized in the second part of this review.

## Biosynthetic pathways of NM

Although some NM can also be found in other brain regions (Zecca et al. 2008b), most NM in the human brain is contained in cell bodies of CA neurons in the SN and LC, where it accumulates with aging (Zecca et al. 2004). Chemical degradative analysis supports that DA and its metabolites are responsible for the production of SN-NM, while NE and its metabolites are responsible for the production of LC-NM (Wakamatsu et al. 2015).

**Fig. 1:**
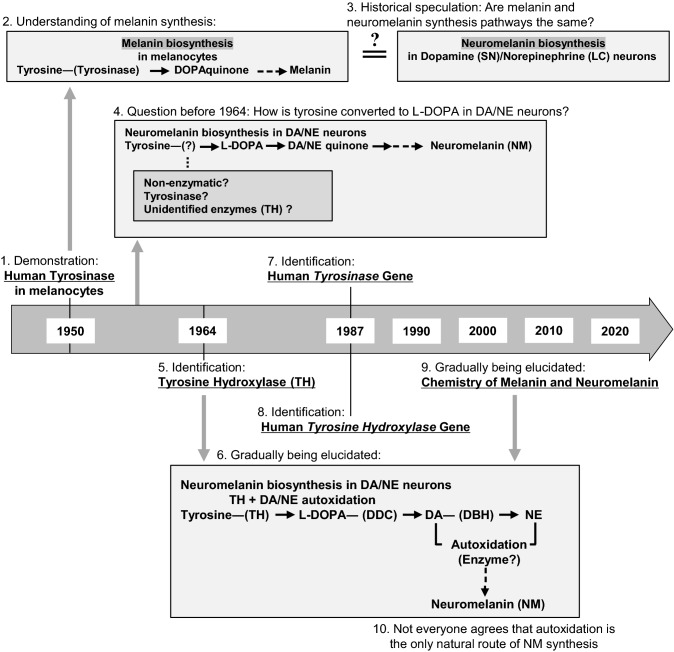
Historic overview of findings and hypotheses in the elucidation of the biosynthesis pathway of neuromelanin (NM). In 1950, Fitzpatrick et al. (1) demonstrated the presence of tyrosinase in melanocytes of peripheral tissues, which (2) fueled the understanding of the role of this enzyme in the biosynthesis of melanin in melanocytes with DOPAquinone as intermediate. (3) Originally, it was speculated that NM was synthesized from tyrosine by a similar pathway as peripheral melanin. (4) However, tyrosinase had not been found in CA neurons, so it was unclear how tyrosine could enter the NM synthesis pathway, and even to date the presence of tyrosinase in DA or NE neurons in the SN and LC of human brain remains elusive. (5) Then, in 1964, Nagatsu et al. identified the enzyme tyrosine hydroxylase form bovine adrenal medulla as an enzyme that catalyzes the conversion of tyrosine to L-DOPA. This finding contributed to (6) the gradual elucidation of the NM biosynthesis pathway which continues to this day. In 1987, the human genes for both tyrosinase (7) (Kwon et al. 1987) and tyrosinase hydroxylase (8) (Grima et al. 1987; Kaneda et al. 1987; Kobayashi et al. 1987) were identified. (9) More details of the chemistry of melanin and NM keep gradually being revealed until this day. (10) Autoxidation of DA or NE is commonly believed to be the (major) route of how these two molecules can enter the NM synthesis pathway, but some authors believe that (also) enzymatic routes are of importance (e.g., Carballo-Carbajal et al. 2019)

A historical overview of discoveries relevant to the biosynthesis pathway of NM is shown in Fig. 1. Melanin synthesis in melanocytes in peripheral tissues such as skin and hair was found to involve conversion by tyrosinase of tyrosine to a highly reactive metabolite of L-3,4-dihydroxyphenylalanine (L-DOPA), namely DOPAquinone (DQ), in the 1940~1950s (Fitzpatrick et al. 1950; Lai et al. 2018; Wakamatsu et al. 2021). The enzyme tyrosinase was first identified near the end of the nineteenth century and has been studied since the first half of the twentieth century. Since the chemical structure of NM was initially assumed to be similar to that of melanin in melanocytes, NM in the SN—which is visible with the naked eye—was also assumed to be synthesized from tyrosine involving tyrosinase. However, whereas tyrosinase was detected in melanocytes of peripheral tissues such as skin (Fitzpatrick et al. 1950), this enzyme could not be found in the SN, posing a critical puzzling question regarding NM biosynthesis.

In 1964, a new enzyme, tyrosine hydroxylase (TH; tyrosine 3-monooxygenase), was found in CA cells, in a study at the National Institutes of Health, Bethesda (Nagatsu et al. 1964). That study also revealed by isotope-tracer experiments that DA and NE are synthesized from tyrosine, and concluded that TH can catalyze the first step in this process by converting tyrosine to L-DOPA (Nagatsu et al. 1964). Interestingly, banana plants synthesize DA from tyrosine by a tyrosinase-like enzyme and do not contain TH (Nagatsu et al. 1972).

A comparison of properties of tyrosinase and TH is shown in Table 1. The human genes for tyrosinase and TH are both situated in chromosome 11. Tyrosinase is expressed in melanocytes in peripheral melanin-containing tissues, such as skin and hair. TH is expressed only in CA (DA, NE, and epinephrine)-producing cells, i.e., CA neurons in the brain, NE neurons in the peripheral sympathetic nerves, and epinephrine and NE cells in the adrenal medulla (Nagatsu 2006). Tyrosinase in melanocytes in peripheral tissues is a copper-containing monomer glycoprotein and oxidizes tyrosine directly to DOPAquinone (DQ; Wakamatsu et al. 2021).

**Table 1:** Comparison between the properties of human tyrosinase and human tyrosine hydroxylase (TH)

	Tyrosinase	Tyrosine Hydroxylase (Tyrosine 3-monooxygenase)
Enzyme (Human)	EC 1.14.18.1 L-tyrosine, L-DOPA: oxygen oxidoreductase	EC 1.14.16.2 L-tyrosine, tetrahydrobiopterin: oxygen oxidoreductase (3-hydroxylating)
Chromosome Exons	11q14.3; 5 Exons	11p15.5; 14Exons
Protein Mr	Glycoprotein Monomer ~ 67,000	Not Glycoprotein Homotetramer ~ 60,000 × 4 Alternative mRNA splicing from a single gene (Internal donor sites and cassette) hTH1 (477aa, ~ 55,000); hTH2 (501aa, ~ 56,000) hTH3 (524aa, ~ 58,000); hTH4 (528aa, ~ 59,000)
Reaction	Tyrosine $\to$ DOPAquinone	Tyrosine $\to$ L-DOPA
Cofactors	2Cu, L-DOPA	Fe × 4 Tetrahydrobiopterin (BH4) × 4
Cells	Melanocytes	Catecholamine neurons (DA neurons: SN; NE neurons: LC) (Dopamine, Norepinephrine, and Epinephrine cells)
Tissues	Skin, Hair, Retina	Brain (Dopamine, Norepinephrine, Epinephrine neurons) Sympathetic nerves (Norepinephrine neurons) Adrenal medulla (Epinephrine cells, Norepinephrine cells)
Metabolic pathway	**Key enzyme** in melanin **B**iosynthesis (M)	Involvement in biosynthesis of CA-derived precursors of neuromelanin (NM)

The structures of human tyrosinase and the tyrosinase-related proteins 1 and 2 (TYRP1 and TYRP2), which are two other enzymes with an important role in the peripheral melanin biosynthetic pathway, were determined by cDNA cloning and biochemical analyses (Kwon et al. 1987; Hearing 1987; Shibahara et al. 1988; Lai et al. 2018). The cDNA sequence of human TH (hTH) was determined in 1987 (Grima et al. 1987; Kaneda et al. 1987; Kobayashi et al. 1987, 1988; O’Malley et al. 1987). Human TH is an iron-containing tetrahydrobiopterin (BH4)-requiring homo-tetramer with four isoforms (hTH1 ~ hTH4) produced by alternative mRNA splicing that differ in the amino acid sequences of their *N*-terminal regions (Grima et al. 1987; Kaneda et al. 1987; Kobayashi et al. 1987, 1988; O’Malley et al. 1987; Lewis et al. 1993; Nagatsu et al. 2019; Bueno-Carrasco et al. 2022).

Expression and distribution of the four isoforms of hTH in human brains were shown by immunochemistry (Lewis et al. 1993; Haycock 2002). The isoforms hTH1 and hTH2 are the major TH isoforms in the human SN and their mRNAs are markedly decreased in the Parkinsonian SN (Ichinose et al. 1994). Monkeys (non-human primates) such as chimpanzee and gorilla express monkey TH1 (mTH1) and monkey TH2 (mTH2), corresponding to hTH1 and hTH2 (Ichikawa et al. 1990; Ichinose et al. 1993). Expression of two isoforms of mTH was reported in macaque monkey brains (Lewis et al. 1994; Haycock 2002). Both mTH1 and mTH2 were found to be markedly decreased in the SN of 1-methyl-4-phenyl-1,2,3,6-tetrahydropyridine (MPTP)-induced PD monkeys (*Macaca fascicularis*; Ohye et al. 1995). TH proteins common in most non-primate mammals, such as rats and mice, correspond to hTH1, the shortest hTH isoform (Lamouroux et al. 1982; Iwata et al. 1992).

DOPA formed by TH is immediately decarboxylated by aromatic L-amino acid decarboxylase (AADC; also called DOPA decarboxylase, DDC) to DA, which is immediately transported into synaptic vesicles by vesicular monoamine transporter 2 (VMAT2) in a stable form (Nagatsu and Stjärne 1998). The activity of VMAT2 is believed to be very important for determining the levels of free DA in the cytoplasm (Segura-Aguilar et al. 2019). Remaining free DA in the cytoplasm is oxidized (deaminated) by monoamine oxidase (MAO) at mitochondrial outer membranes to form 3,4-dihydroxyphenylacetaldehyde (Kopin 1985; Eisenhofer et al. 2004), although additional enzymes are involved in minor degradation pathways of DA. The rate of NM synthesis is presumably very slow compared to DA synthesis. Early in the NM synthesis pathway, free DA remaining in the cytoplasm is converted to DAquinone (DAQ), by autoxidation with iron or copper as catalysts, or maybe also by tyrosinase if it is present (the latter being very debatable). Thus, an increase in cytoplasmic DA appears able to increase the formation of NM (Sulzer et al. 2000). As the levels of free DA in the cytoplasm are determined by the activities of TH, AADC (DDC), MAO, and VMAT2, and other enzymes variably involved in DA pathways, all these activities also affect the biosynthesis of NM.

DA in the cytoplasm can lead to toxicity, because it can easily be oxidized to DAQ, causing oxidative stress and damage through protein-quinone modifications (Monzani et al. 2019) finally leading to mitochondrial and lysosomal dysfunction (Burbulla et al. 2017). It was reported that recombinant expression of tyrosinase, the gene of which is not associated with PD, exacerbates DA toxicity, probably by promoting the oxidation of DA to DAQ (Greggio et al. 2005). A similar result was reported in a transgenic human neuronal cell line in which tyrosinase expression could be induced, serving as an in vitro model of PD (Hasegawa 2010).

**Fig. 2:**
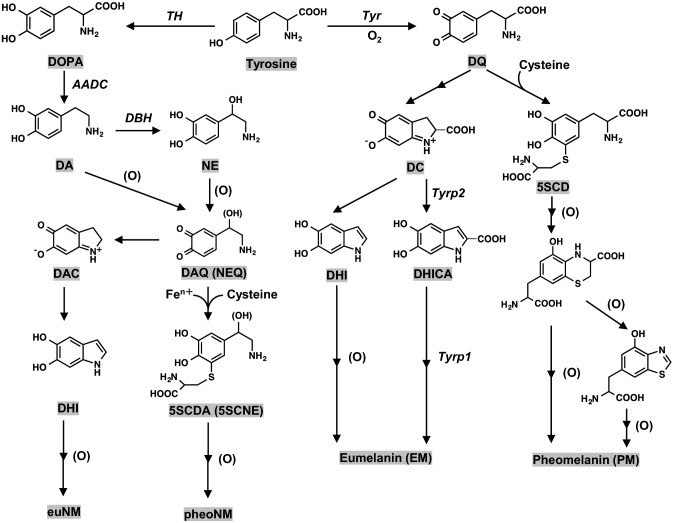
Biosynthesis pathways of eumelanin (EM) and pheomelanin (PM) in melanocytes in peripheral tissues (i.e., skin and hair) and of eumelanic portion of NM (euNM) and pheomelanic portion of NM (pheNM) in human brain. The eumelanic pigments are usually darker and brown or black in color, while the pheomelanic pigments are lighter and more yellowish/reddish in color. Eumelanin and pheomelanin differ not only in color but also in their redox, metal chelating, and free radical scavenging properties. Eumelanin is an antioxidant, more stable, and photoprotective, while pheomelanin is more prone to photodegradation and can act as a pro-oxidant by either reducing antioxidants or generating reactive oxygen species (d'Ischia et al. 2013). For brain NM, the scheme represents the initial steps of the growing melanic portion of NM, a very complex pathway that later involves more proteins, lipid and metals at various steps and the biogenesis of NM-organelles. DAQ: DAquinone; NEQ: NEquinone; DAC: DAchrome; DHI: 5,6-dihydroxyindole; 5SCDA: 5-*S*-cysteinyldopamine; 5SCNE: 5-*S*-cysteinylnorepinephrine; DQ: DOPAquinone; DC: DOPAchrome; DHICA: 5,6-dihydroxyindole-2-carboxylic acid; 5SCD: 5-*S*-cysteinyldopa; Tyr: tyrosine; Tyrp1: tyrosine-related protein 1; Tyrp2: tyrosine-related protein 2. Enzyme names are shown in italic for the sake of clarity. (O): oxidant (Reference: Nagatsu et al.^2022^). Although in mice Tyrp1 acts as a DHICA oxidase as indicated in the figure, its human homolog may not act in the same way and its precise enzymatic function is not yet clear (Boissy et al. 1998)

We could not identify tyrosinase in the human SN by a sensitive immunohistochemistry using anti-human tyrosinase antibodies (Ikemoto et al. 1998), and the absence of tyrosinase in the human SN was also indicated by several other reports using different techniques (Tribl et al. 2007; Plum et al. 2016; Zucca et al. 2018). In melanin biosynthesis in human skin, TYRP1 and TYRP2 are required after tyrosinase reaction (Fig. 2), but neither TYRP1 nor TYRP2 has been identified yet in NM biosynthesis. Furthermore, in two albino individuals with probably a genetic defect in tyrosinase, NM amounts in the brain were found to be normal, which also suggests the absence of a role of the enzyme in human brain NM synthesis (Foley and Baxter 1958). In addition, mass spectrometry analyses of the proteome of different kinds of isolated NM samples from human SN could not find tyrosinase but did find TH (Plum et al. 2016; Zucca et al. 2018; Wulf et al. 2022a).

On the other hand, there have been several reports that tyrosinase mRNA, protein, and activity exist at very low levels in the SN of human and murine brains (Xu et al. 1997; Tief et al. 1998; Greggio et al. 2005). Moreover, in vitro, synthetic “NM-like” pigment can be made using tyrosinase that oxidizes DA and NE to DAQ and NEquinone (NEQ), respectively, as pathway intermediates (Wakamatsu et al. 2012). Also in vivo, artificial expression of human tyrosinase in rat SN leads to the formation of NM-like pigment in DA neurons (Carballo-Carbajal et al. 2019). In summary, if there would be sufficient tyrosinase in human CA neurons, which is very doubtful, then it probably would be one of the factors contributing to pigment synthesis by providing an additional pathway besides CA autoxidation. However, it is critical to note that—as far as investigated—the structures of “tyrosinase-made” melanins are characterized by an aromatic stacking organization, as demonstrated by X-ray spectra, that is not found in natural NM, and that the different structures may affect functional properties (Zecca et al. 2008b).

Because (i) tyrosinase is not convincingly found in the human SN, (ii) TH, unlike tyrosinase, does not oxidize DOPA, DA and NE, and (iii) free DA and NE in the cytoplasm are easily autoxidized to DAQ and NEQ, the pathway for the biosynthesis of the melanic portion of NM in human brain appears to be (Fig. 2) (Nagatsu et al. 2022): (1) DA and NE are synthesized from tyrosine: tyrosine – (TH) → L-DOPA – (AADC/DDC) → DA – (dopamine-β-hydroxylase/DBH) → NE in SN and LC neurons, respectively; (2) DA and NE are nonenzymatically autoxidized to DAQ and NEQ probably with iron or copper as catalysts (Zecca et al. 2004; Zucca et al. 2017; Sun et al. 2018; Monzani et al. 2019; Ito et al. 2022); and (3) in the presence of cysteine, DAQ or NEQ is converted to 5-*S*-cysteinyl DA (5SCDA) or 5-*S*-cysteinyl NE (5SCNE) (Fornstedt et al. 1990; Shen et al. 1998) and further oxidized to pheomelanic NM (pheoNM), whereas in the absence of cysteine, DAQ or NEQ is converted to DAchrome (DAC) or NEchrome (NEC) through cyclization, followed by conversion to 5,6-dihydroxyindole (DHI) through tautomerization (Bisaglia et al. 2007) and eventually undergoing further oxidation and polymerization to eumelanic NM (euNM). This biosynthetic process can be considered a mixed-type melanogenesis of eumelanin (EM) and pheomelanin (PM) (Zecca et al. 2008b; Monzani et al. 2019; Wakamatsu et al. 2021; Zucca et al. 2023).

Thus, TH in CA cells initiates both the biosynthesis of CAs as neurotransmitters and the biosynthesis of NM via DA and NE. That DA and NE synthesized by TH are autoxidized to DAQ and NEQ, likely through metal-catalyzed oxidation, and further to euNM and pheoNM is a viewpoint in agreement with earlier proposals (e.g., Fornstedt et al. 1986; Engelen et al. 2012; Zucca et al. 2017; Wulf et al. 2022a).

Very recently (Cai et al. 2023), we found increased pheomelanin and reduced eumelanin components in NM of PD brains compared to control subjects, although this may be related to the medication of the sample donors with L-DOPA (levodopa; see below). Furthermore, we found using neural cell cultures that synthetic DOPA pheomelanin induced neuronal cell death, while synthetic DOPA eumelanin showed no significant effect on cell viability, and believe this may reflect properties of the pheomelanic and eumelanic components of natural NM (Cai et al. 2023).

## Discussion of questions

### Is there a quantitative correlation at the cellular level between NM and neurodegeneration in PD, so that the cells with the most NM die?

Sporadic PD is a disease of the elderly, the life stage in which the NM amounts in SN and LC are high (Zecca et al. 2002, 2004). As it is mostly the NM-containing CA neurons that die in PD, a reasonable hypothesis would be that high NM levels either directly contribute to their death, or reflect the process or condition leading to their death. On the other hand, overall NM amounts in Parkinsonian SN and LC are lower than in controls (Mann and Yates 1983; Zecca et al. 2002), although that may be primarily caused by the dying of NM^+^ neurons (Hirsch et al. 1988, 1989). Four different studies seem to agree that, for each individual CA neuron, high NM concentrations do not immediately or necessarily lead to cell degeneration, but on average promote this process (Mann and Yates 1983; Kastner et al. 1992; Halliday et al. 2005; Carballo-Carbajal et al. 2019)—which implies that NM can only be one of the factors contributing to neurodegeneration in PD (Cebrián et al. 2014; Riederer et al. 2021). Studies differ, however, on the issue of whether the NM amounts per NM^+^ CA neuron are higher in PD patients than in healthy elderly.

**Fig. 3:**
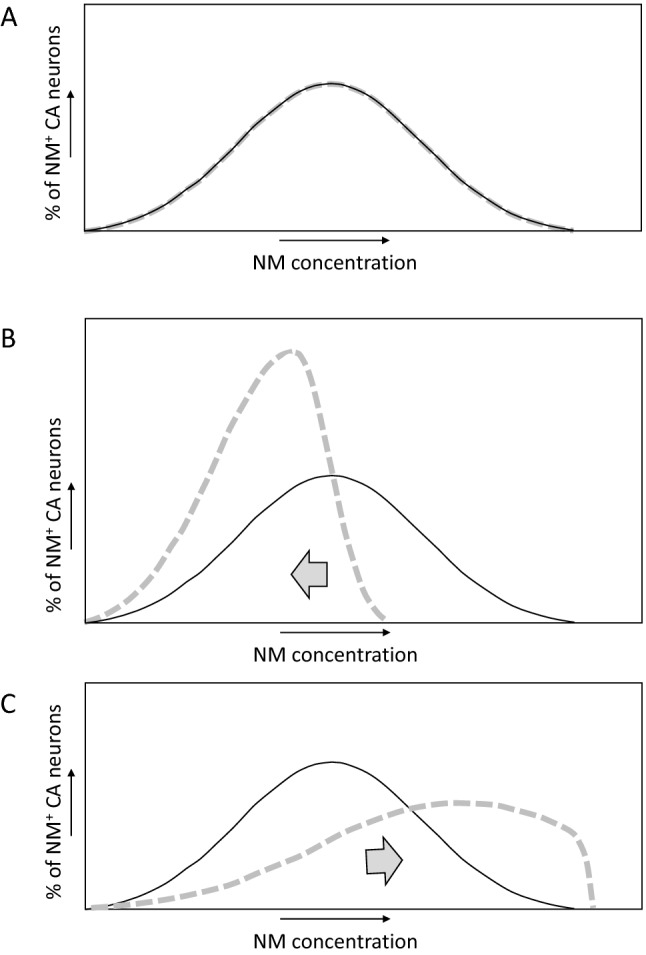
Simplified graph figures of how, theoretically, the distribution of cellular NM concentrations among NM^+^ CA neurons in the SN or LC may compare between PD patients (dashed gray line) and controls (black line) if **A** there is no difference in PD neurodegeneration related to NM concentrations per cell, **B** if in PD the NM concentrations per cell are not higher than in controls and if in PD those cells with a higher NM content are more likely to die, and **C** if in PD the NM concentrations per cell are higher than in controls and if in PD those cells with a higher NM concentration are more likely to die. It is not precisely known how NM concentrations are distributed per CA neurons, and the shapes of the graphs in this figure are partly speculative; the figure is only meant as a visual aid in a theoretical discussion

Theoretically, if the sensitivity to PD of CA neurons would be caused by a factor that is quantitatively unrelated to NM, the NM concentrations in the surviving NM^+^ CA neurons would not differ from controls (as shown in the simplified graph figure in Fig. 3A). If, on the other hand, NM concentrations per NM^+^ CA neuron would not be enhanced in PD but be a contributing factor to their death, lower concentrations of NM would be expected in surviving NM^+^ CA neurons than in controls (Fig. 3B). Then again, if higher NM concentrations in CA neurons in PD would be the cause of CA neuron death by surpassing an acceptable threshold level of NM—or be quantitatively correlated with a deadly cause that surpasses a threshold level—higher NM concentrations per NM^+^ CA neuron are expected than in controls (Fig. 3C).

The four mentioned studies that investigated NM concentrations in individual neurons (Mann and Yates 1983; Kastner et al. 1992; Halliday et al. 2005; Carballo-Carbajal et al. 2019) can be divided into two groups:

#### Studies that suggest that NM concentrations per NM^+^ CA neuron are quantitatively associated with cell death but are not higher in PD

• Mann and Yates (1983) found that in NM^+^ neurons of the SN the NM optical density (an estimation of the relative NM amount which is not without technical issues) was, on average, lower per cell in eight PD patients compared to 20 age-matched controls, and that the frequency histograms of these results showed a direction as in our Fig. 3B.

• Kastner et al. (1992) found in PD patients that the mean optical density of NM per remaining NM^+^ cell in the mesencephalon (from SN and ventral tegmental area) was 6% less than in controls (from four individuals each), and that frequency histograms of the optical density per cell moved slightly into the direction shown in our Fig. 3B. For the SN in PD patients, they found increased percentages of lightly melanized neurons among the NM^+^ neurons in the pars and lateralis, which contributed to this shift.

#### Studies that suggest that enhanced NM concentrations per cell contribute to PD

• Halliday et al. (2005) criticized studies as by Kastner et al. (1992) and argued that specific subpopulations of NM^+^ neurons should be examined more closely. They found that degenerating NM^+^ neurons in Parkinsonian SN A9 region had lower NM optical densities than healthy NM^+^ SN A9 neurons in controls. On the other hand, in morphologically normal NM^+^ neurons in Parkinsonian SN A9 region, NM optical densities were increased. The authors proposed that increased cellular NM optical densities precede neurodegeneration, the latter being accompanied by an accumulation of Lewy bodies and a dispersement of NM within the cell, resulting in a lower optical density of NM.

• Carballo-Carbajal et al. (2019) reported that when comparing PD patients with age-matched controls, on average, the intracellular NM optical density in SNpc NM^+^ neurons was higher in PD subjects. Moreover, they found that expression of human tyrosinase in rat or mouse SN using an adeno-associated viral vector resulted in a gradual—taking multiple months—production of NM-like pigment in vesicles within nigral DA neurons, up to levels reached in elderly humans. They found that when in these animals, on average, the intracellular pigment optical density in SNpc NM^+^ neurons was as high as in PD patients, this was associated with a PD phenotype, including hypokinesia, Lewy body-like formation, and nigrostriatal neurodegeneration. Their data in both humans and rodent models represent a distribution in the direction of our Fig. 3C. However, as mentioned above, it is of note that, as far as investigated, pigment produced through the tyrosinase route was shown to be structurally different from natural NM that results from autoxidation of DA and related metabolites (Zecca et al. 2008b).

In summary, looking at the combined four studies here listed (Mann and Yates 1983; Kastner et al. 1992; Halliday et al. 2005; Carballo-Carbajal et al. 2019), we believe that it has been credibly shown that in PD there is a quantitative association between the concentration of NM in CA neurons and their vulnerability. Thus, NM is not just a binary “present or absent” marker for PD vulnerability. However, the reports appear to be conflicting on whether PD is associated with higher or lower concentrations of NM per surviving NM^+^ CA neurons compared to non-PD controls, and future studies should clarify this.

It is important to realize that studies of the last 30 years reported that specific cellular conditions make NM protective—by scavenging and sequestering of toxic molecules—or toxic for neurons (Zecca and Swartz 1993; Double et al. 2002; Zucca et al. 2017). Thus, quantitative associations of NM with cell degeneration are complicated, and in some conditions increased NM may only be an indicator of toxic factors against which it tries to protect. The protective versus toxic roles of NM are an ongoing debate.

### Does treatment of PD patients with L-DOPA lead to increased concentrations of NM in their CA neurons?

L-DOPA is also known as “levodopa” and, as a drug, it has been remarkably successful in reducing PD symptoms since its introduction in the 1960s (Hornykiewicz 2010). Typically, by oral route, PD patients are administered large amounts of levodopa (up to more than a gram per day), which has the advantage over DA that it can cross the blood–brain barrier. Levodopa treatment leads to considerable increases of DA in the striatum (Lloyd et al. 1975; Abercrombie et al. 1990).

If this levodopa conversion to DA would take place in DA neurons of the SN, higher NM concentrations may be expected in the SN of patients treated with levodopa. For example, the group of Luigi Zecca induced NM production in rat SN primary cell cultures and PC12 cell line by exposing them to levodopa, which was taken up by the cells and rapidly converted to DA in the cytosol (Sulzer et al. 2000; Segura-Aguilar et al. 2019). Moreover, they found that if in these cells the excess DA was transferred from the cytosol by transgenic overexpression of VMAT2—which sequesters DA into synaptic vesicles—NM production was drastically reduced, providing evidence for the involvement of cytosolic DA in NM biosynthesis.

Thus, where in the brain is the drug levodopa converted to DA for elevating DA levels in the striatum? In the brain, at least under conditions in which a considerable percentage of DA SN neurons has degenerated, serotonergic neurons seem to be involved in the bulk of this levodopa-to-DA conversion as shown by the effect of their lesion in a hemiparkinsonian rat model (Tanaka et al. 1999; see also Chagraoui et al. 2019). Serotonergic neurons innervating the striatum from the raphe nuclei are probably major contributors to the striatal DA increases after levodopa treatment (Reed et al. 2012). Serotonergic neurons appear to take up levodopa through L-amino acid transporter (LAAT), convert it to DA using AADC, and store it in vesicles until release upon cell activation (Ng et al. 1970; Gantz et al. 2015; Mosharov et al. 2015). At least in vitro, DA midbrain neurons seem to be able to do the same thing (Sulzer et al. 2000; Mosharov et al. 2009), and it is assumed that also in levodopa-treated patients this process takes place (Reed et al. 2012; Mosharov et al. 2015). However, to the best of our knowledge, there appears to be no direct evidence for levodopa treatment significantly increasing the DA or NM amounts in SN DA neurons in PD patients. For example, the Mann and Yates (1983) study did not find significant differences in NM optical densities in NM^+^ cells between four PD patients that had been treated 1–5 years with levodopa and four PD patients that had not been treated. Furthermore, although in vitro the higher DA levels in DA neurons induced by exogenous levodopa were accompanied by increased cell vulnerability (Mosharov et al. 2009), likely through different pathways related to toxic metabolites of DA (Monzani et al. 2019), there is no evidence that levodopa treatment of PD patients increases DA neuron degeneration (reviewed in Simuni and Stern 1999; Fahn 2006). Typically, in vitro experiments use higher levodopa concentrations than those reached in the brain of levodopa-treated patients, and when using cell lines the experiments are usually performed in the absence of glial cells, which may help explain the differences.

Regarding the question of whether DA or NM can be toxic for DA neurons in vivo, it is sometimes debated that the success of levodopa treatment of PD patients argues against such toxicity. However, before this can be considered a serious argument, it should probably first be proven that levodopa treatment increases the DA or NM concentrations in the remaining DA neurons of PD patients indeed.

Very recently, we found that the ratio of pheomelanic to eumelanic components is higher in NM of Parkinsonian SN than in controls, and this may be an effect of levodopa treatment (Cai et al. 2023). However, such a conclusion would need comparison with samples from PD patients that had not been treated with levodopa, and our study was not developed like that. Currently, it cannot be known whether the observed shift in the “pheoNM/euNM” ratio (Cai et al. 2023) derives from the disease or the levodopa medication.

Chronic levodopa therapy in PD patients tends to be accompanied by rapid variations in treatment efficacy (on–off fluctuations) and involuntary movements (levodopa-induced dyskinesia). These adverse symptoms have been proposed to be importantly caused by an increasing reliance on the levodopa-to-DA conversion by serotonergic neurons while these cells do not have the proper molecular mechanism for DA release management and feedback control; however, many questions remain (Reed et al. 2012; Mosharov et al. 2015; Fabbrini and Guerra 2021). Notably, in a study where levodopa therapy was initiated years after onset of PD, it was reported that motor fluctuations and dyskinesias were not associated with the duration of levodopa treatment, but with duration of disease and higher dosage of levodopa (Cilia et al. 2014).

### Does an active lifestyle increase CA secretion and, therefore, lower NM production?

As shown in the above-mentioned study on VMAT2 expression by Sulzer et al. (2000), NM synthesis can be stimulated by increasing cytosolic DA concentrations, while its biosynthesis can be reduced by decreasing and secreting DA. The DA from SN neurons is released in the striatum when a person engages in motor activities, and NE from LC neurons is released upon excitement/stress. For example, exercise was found to increase plasma levels of NE (Hackney 2006) and striatum levels of DA (Bastioli et al. 2022). Therefore, a logical question is whether an active lifestyle helps to keep NM concentrations down. However, we are not aware of studies that compared NM amounts per lifestyle group among non-PD individuals, although there are studies on how exercise may help PD patients to avoid further degeneration of NM^+^ neurons (Hackney et al. 2020).

### What is the role of iron?

Inside CA neuron cell bodies of LC and SN, NM pigment is contained in double-membraned vesicles (organelles) in which it is associated with metal ions, lipids, and proteins (Zecca et al. 2000, 2004, 2008b; Double et al. 2003; Fedorow et al. 2005a; Engelen et al. 2012; Zucca et al. 2018). Among these metal ions, iron is the most abundant one (Zecca et al. 2004), and the paramagnetic iron accumulations into NM (Zecca and Swartz 1993; Capucciati et al. 2022) are so pronounced that NM-iron complex is detectable by magnetic resonance imaging (Sasaki et al. 2006; Cassidy et al. 2019). In the SN of the elderly the iron concentration increases, but not as dramatic as NM content, while in the LC of the elderly the iron concentrations appear not to be increased (Zecca et al. 2004). In PD patients, there is an even considerably stronger increase of iron in the SN, while not in several other brain regions, suggesting a causative relationship between iron and PD (Dexter et al. 1987; Sofic et al. 1988; Riederer et al. 1989; Jellinger et al. 1992; Youdim et al. 1993; Wang et al. 2016). The functional effects of the associations between iron and NM, and their involvement in PD progression, are topics of debate.

Iron is a transition metal that in biological materials can reversibly switch between Fe^2+^ (ferrous iron) and Fe^3+^ (ferric iron) states and is used as a critical component in many enzymes for electron transfer reactions (Dlouhy and Outten 2013). However, if there is a surplus of iron, the iron in the labile iron pool (chelatable and redox-active iron in complexes of low stability) increases and the now uncontrolled reducing power of Fe^2+^ has a variety of toxic effects through the generation of reactive oxygen species (ROS; Brissot et al. 2012). In Parkinsonian SN, the oxidative stress caused by a surplus of iron is believed to directly contribute to PD through a variety of processes such as, for example, membrane lipid peroxidation (Riederer et al. 1989, 2021; Ben-Shachar et al. 1991; Trist et al. 2019).

As mentioned above, iron plays a role in the synthesis of NM by catalyzing autoxidation of DA to DAQ (Fig. 2). In this process, Fe^3+^ ions coordinate with the catechol skeleton of DA, resulting in electron transfer to produce quinones and Fe^2+^ ions (Halliwell et al. 1989; Zucca et al. 2017). As for the NM polymeric structures, Ben-Shachar et al (1991) found that there is a high-affinity (*K*_D_ = 13 nM) and a lower affinity (*K*_D_ = 200 nM) binding site for iron on synthetic DA melanin, although it should be kept in mind that the structure of this synthetic melanin is different from human NM (Zecca et al. 2008b). High- and low-affinity binding sites were later also found for natural human NM (Double et al. 2003). The sites are reported to be formed by a high-affinity multinuclear iron cluster in which redox-inactive Fe^3+^ is bound by oxo and/or hydroxo bridges and is surrounded by catechol groups of NM, and a lower affinity mononuclear iron site in which a single iron ion is six-coordinated by oxygen atoms of catechol moieties, and possibly by water or hydroxo groups (Zecca et al. 2001; Zucca et al. 2017; Monzani et al. 2019). The two different iron binding sites of NM have been recently reviewed both in natural NM and in new synthetic models (Zucca et al. 2023). It was speculated that the low-affinity binding site is only occupied in case of iron overload, when the high-affinity centers are saturated (Zecca et al. 2008c; Zucca et al. 2017, 2018; Monzani et al. 2019). Such a model fits well with the finding by Faucheux et al. (2003) that the amount of redox-active iron per amount of NM is increased in Parkinsonian SN.

The chelation of iron (along with other potentially toxic metals) by NM and its incorporation in vesicles are believed to help protect the cells against iron-mediated oxidative stress, while simultaneously the iron bound to NM is believed to form a potential reservoir of iron toxicity that may help explain why NM is associated with neurodegeneration in PD (Ben-Shachar and Youdim 1990; Ben-Shachar et al. 1991; Jellinger et al. 1992; Fedorow et al. 2005a; Biesemeier et al. 2016; Zucca et al. 2017; Riederer et al. 2021). This dual model is corroborated by in vitro experiments, in which synthetic DA melanin (Pilas et al. 1988; Ben-Shachar et al. 1991; Zecca et al. 2008c) and human NM (Zecca et al. 2008c) were found to either reduce or enhance iron-mediated oxidation processes, depending on conditions. However, how those results translate to the situation in CA neurons where NM is normally shielded from the cytoplasm by a double membrane is less clear. Lopiano et al. (2000) and Bolzoni et al. (2002) found that NM samples from PD patients showed lower magnetic values than from non-PD controls, suggesting that less iron is bound, although Bolzoni et al. (2002) described technical difficulties and marked the results as “preliminary.” Nevertheless, these two studies may be interpreted as NM in PD being toxic by releasing iron to below-normal concentrations per NM unit (e.g., Fedorow et al. 2005a). On the other hand, as described above, Zecca and co-workers have proposed a “saturation” model postulating that in PD the increased labile and redox-active pool of iron bound to the low-affinity binding site of NM can contribute to oxidative stress. Given the absence of perfect animal models for PD, it may take long before the interaction between NM and iron will be perfectly understood and further experimental as well as human post mortem studies are necessary.

### What is the source of the cysteine used in NM synthesis?

Zecca and co-workers showed that cysteine in proteins rather than free cysteine contributes to pheoNM biosynthesis (Ferrari et al. 2017; Monzani et al. 2019; Zucca et al. 2023). Oxidized DA binds to protein through cysteine residues of protein, but it is still unknown how conjugates are oxidized subsequently. Wakamatsu et al. (2019) examined in detail the NM biosynthesis pathway by oxidation of DA with peptides and proteins using spectrophotometric and high-performance liquid chromatography (HPLC) methods. They prepared a variety of thiol-bound DA complexes and showed that DA binds via a cysteine residue of proteins and that the efficacy of binding depends on the structural features of the proteins. The results also suggest that oxidatively modified DA-protein conjugates produced in the brain possess a potent pro-oxidant activity, which may cause neurodegeneration through the production of ROS and the depletion of antioxidants (Wakamatsu et al. 2019).

### Which other molecules are found in NM-organelles?

Besides NM itself, NM-organelles contain proteins and lipid bodies. The double membrane of NM-organelles (Duffy and Tennyson 1965; Sulzer et al. 2000; Zecca et al. 2008b; Monzani et al. 2019) and their inclusion of some autophagosome marker proteins like microtubule-associated proteins 1A/1B light chain 3B (MAP1LC3B) and the autophagic adaptor sequestosome-1 (SQSTM1) support a macroautophagic origin, and it is believed that NM-organelles originate from autophagosomes engulfing NM precursors, lipids, and proteins from the cytosol (Zucca et al. 2018). Besides the autophagosome signature, NM-organelles also have a pronounced lysosome signature. Lysosomal enzymes rich in NM-organelles are peptidases, sulfatases, and esterases, while lipases and glycosylases occur in lower amounts (Tribl et al 2005, 2006; Plum et al. 2016; Zucca et al 2018; Wulf et al. 2022a). Therefore, NM-organelle origin probably involved fusions between autophagosomal and lysosomal vesicles (Zucca et al. 2018). NM-organelles also contain proteins like alpha-crystallin B chain, heat shock protein HSP 90-alpha, tubulin polymerization-promoting protein (TPPP), glycoprotein nonmetastatic melanoma protein (GPNMB), and ubiquitins, which are proteins that likely play a role in aggregation and degradation processes involved in NM-organelle formation; this suggests a role for accumulation of excess molecules by this organelle when the ubiquitin–proteasome system is inadequate (see the model in Zucca et al. 2018). Furthermore, high abundances of proteins of the 40S ribosomal subunit in combination with several eukaryotic translation initiation factors (eIF) and RNA-binding proteins led to the proposal of a potential link between NM-organelles and stress granules (Wulf et al. 2022a). Some other notable proteins that are associated with NM-organelles are α-synuclein and major histocompatibility complex (MHC) class I proteins (Zucca et al. 2018; Wulf et al. 2022a, b). MHC class I function was shown to render CA neurons vulnerable to attacks by cytotoxic T cells and may contribute to the death of NM^+^ CA neurons (Cebrián et al. 2014). The major lipid components in the lipid bodies of NM-organelles are dolichols and dolichoic acids (Fedorow et al. 2005b; Zucca et al. 2018).

## Conclusion

NM in CA neurons in the SN and LC appears to be, at least predominantly, synthesized by a pathway involving tyrosine hydroxylase and iron/copper-mediated autoxidation, and it accumulates in organelles. In Parkinsonian SN and LC, the NM^+^ neurons preferentially die, especially those with higher NM contents. Although NM is quantitatively correlated with neurodegeneration and may be a contributing factor, it cannot be the only reason. The role of NM in neurodegeneration is complex, as it appears to have both protective and toxic roles, depending on the cellular conditions. Questions remain as to whether NM in the human brain can also be synthesized by a tyrosinase-involving pathway—which authors of the present article deem unproven and unable to make a major contribution—and whether NM concentrations per cell are higher in PD patients. It seems worthwhile to consider whether regular exercise might reduce NM synthesis and thereby help to prevent neurodegeneration.

## Data Availability

Not applicable.
